# Case Report: Ménière's Disease-Like Symptoms in 22q11.2 Deletion Syndrome

**DOI:** 10.3389/fneur.2021.690078

**Published:** 2021-06-18

**Authors:** Kwang-Dong Choi, Jeong-Yeon Kim, Seo-Young Choi, Eun Hye Oh, Hyun-Min Lee, Jieun Roh, Jae-Hwan Choi

**Affiliations:** ^1^Department of Neurology, Pusan National University School of Medicine and Biomedical Research Institute, Pusan National University Hospital, Busan, South Korea; ^2^Department of Neurology, Pusan National University School of Medicine, Research Institute for Convergence of Biomedical Science and Technology, Pusan National University Yangsan Hospital, Yangsan, South Korea; ^3^Department of Otorhinolaryngology, Pusan National University Yangsan Hospital, Yangsan, South Korea; ^4^Department of Radiology, Pusan National University Yangsan Hospital, Yangsan, South Korea

**Keywords:** 22q112 deletion syndrome, Ménière's disease, endolymphatic hydrops, case report, vertigo, sensorineural hearing loss

## Abstract

The 22q11.2 deletion syndrome (22q11.2DS), caused by a microdeletion on the long arm of chromosome 22, is characterized by congenital heart disease, hypoparathyroidism, immunodeficiency, developmental delay, and velopharyngeal insufficiency. Anatomic malformations of the middle and inner ears are frequently present, leading to high prevalence of hearing impairment. We present a first case of 22q11.2DS showing fluctuating hearing loss with recurrent vertigo attacks, resembling Ménière's disease. A 38-year-old male known to have 22q11.2DS developed recurrent vertigo, tinnitus, and fluctuating hearing loss in the left ear during a 10-year follow-up period. During vertigo attack, he had spontaneous left-beating nystagmus with downbeat components, but bithermal caloric and video head impulse tests showed normal vestibulo-ocular reflex functions. Sequential pure tone audiograms demonstrated fluctuating sensorineural hearing loss (SNHL) in both ears, which finally progressed to permanent hearing loss in the left ear. Computed tomography imaging of the temporal bone exhibited bilaterally malformed lateral semicircular canals, and delayed 3D-FLAIR sequences revealed cochlear endolymphatic hydrops with dilation of the scala media in the left ear. This case shows that acute vertigo with SNHL can be one of the audiovestibular presentations in 22q11.2DS caused by disturbance of endolymphatic flow.

## Introduction

The 22q11.2 deletion syndrome (22q11.2DS), also known as DiGeorge syndrome, is caused by a microdeletion on the long arm of chromosome 22 (1). Patients with 22q11.2DS exhibit highly variable phenotypes that include congenital heart disease, hypoparathyroidism, immunodeficiency, developmental delay, and velopharyngeal insufficiency. Anatomic malformations of the middle and inner ears are frequently present, leading to high prevalence of hearing impairment (2, 3). While abnormal morphology of the vestibule and the lateral semicircular canal (LSCC) has been also reported, most patients do not present with vestibular symptoms (4). In this report, we present a first case of 22q11.2DS showing fluctuating hearing loss with recurrent vertigo attacks, resembling Ménière's disease.

## Case Report

**Figure 1:**
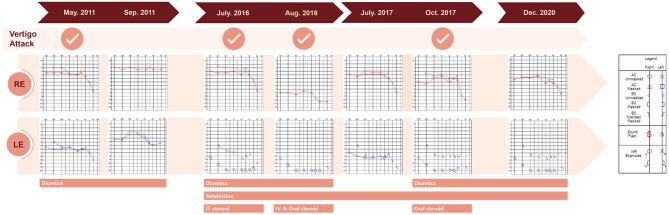
Time course of vertigo attacks, pure tone audiogram results, and treatments. AC, air conduction; BC, bone conduction; IT, intratympanic; IV, intravenous, LE, left ear, NR, no response; RE, right ear.

**Figure 2:**
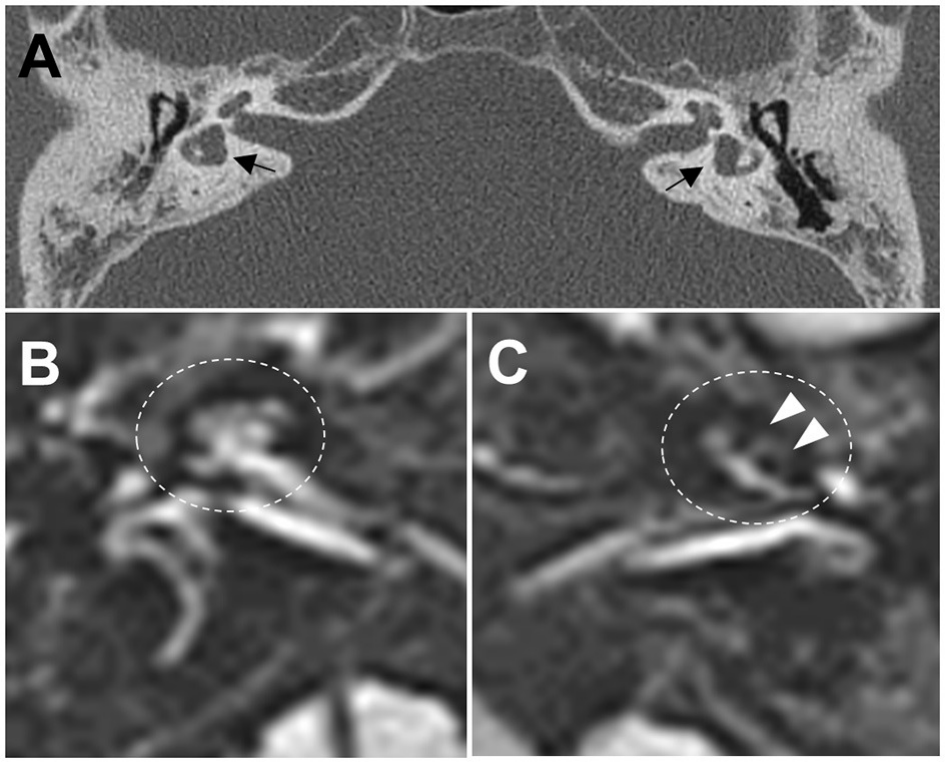
**(A)** Computed tomography images of the temporal bone demonstrate bilateral widening of the vestibules with an abnormally small bony island of lateral semicircular canals (black arrows) and soft tissue densities in the middle ear, and decreased mastoid air cells. **(B,C)** Axial three-dimensional fluid-attenuated inversion recovery images obtained 4 h after intravenous administration of gadolinium reveal cochlear endolymphatic hydrops (EH) with dilation of the scala media in the left ear (**C**, white arrows), but no EH in the right ear **(B)**.

A 38-year-old male known to have 22q11.2DS presented with recurrent vertigo lasting for hours and fluctuating tinnitus in the left ear. He had a history of neonatal seizure, hypocalcemia due to hypoparathyroidism, and intellectual disability, and underwent cardiac surgery due to congenital heart disease (tetralogy of Fallot) at 15 years of age. On examination, the patient showed characteristic facial features of 22q11.2DS such as hypertelorism, a short and broad nose, and a deeply grooved philtrum. There was no spontaneous nystagmus between vertigo attacks, and bedside head impulse and bithermal caloric tests were normal. The pure tone audiogram (PTA) demonstrated sensorineural hearing loss (SNHL) in the left ear (Figure 1). Computed tomography (CT) imaging of the temporal bone revealed bilateral widening of the vestibules, soft tissue densities in the middle ear, and decreased mastoid air cells (Figure 2A). The patient was diagnosed with Ménière's disease, and treated with a low-salt diet and diuretics. The vertigo attacks resolved, and follow-up PTA revealed mild improvement of SNHL in the left ear (Figure 1).

Five years after his initial presentation, the patient developed recurrent vertigo, hearing loss, and tinnitus in the left ear again. A physical examination demonstrated spontaneous left-beating nystagmus with downbeat components that increased during left eccentric gaze. Bithermal caloric tests were normal, and video head impulse tests showed normal vestibulo-ocular reflex (VOR) gains for all six semicircular canals (SCCs). PTA revealed profound SNHL in the left ear (Figure 1). Treatment with diuretics, betahistine, and intratympanic steroid injection to the left ear failed to attenuate the vertigo attacks. A 1-month follow-up PTA revealed additional hearing loss in the right ear without improvement of the left SNHL (Figure 1). The patient received an intravenous steroid injection followed by a tapering dose of oral steroids, resulting in a gradual reduction of the frequency of vertigo attacks. At the 1-year follow-up PTA, there was improvement of hearing loss in both ears, particularly in the right ear (Figure 1). Since the vertigo attacks recurred after stopping diuretics, the patient was maintained on combination therapy with betahistine and diuretics. During a 3-year follow-up period, he remained symptom-free despite the persistence of left SNHL (Figure 1). At the latest follow-up, the patient underwent delayed 3T magnetic resonance imaging (MRI; MAGNETOM Vida, Siemens, Erlangen, Germany) using a 64-channel array head coil, 4 h after intravenous gadolinium (12 ml of gadoterate meglumine, Dotarem; Guerbet, Aulnay-sous-Bois, France) to evaluate the presence of endolymphatic hydrops (EH) (5). We performed the 3D fluid-attenuated inversion recovery (3D-FLAIR) images with the following parameters; field of view (FOV): 160 × 160 mm, repetition time (RT): 7,000 ms, echo time (ET): 303 ms, inversion time (IT): 2,050 ms, matrix size: 256 × 230, flip angle: 120°, number of excitation (NES): 2, and scan time of 6 min 39 s. Axial 3D-FLAIR revealed cochlear EH with dilation of the scala media in the left ear (Figures 2B,C), but no cochlear enhancement or vestibular EH.

## Discussion

Among the affected genes in 22q11.2DS, deletion of *TBX1* is responsible for the main features of the disease, such as heart problems, hypoplasia of the thymus and parathyroid glands, and velopharyngeal insufficiency (6). *TBX1* is also expressed in the otic vesicle and the periotic mesenchyme during development, and was shown to be necessary for the development of the inner ear in mice (7). Thus, homozygous *TBX1* mutant mice developed inner ear malformations that are characterized by an absent or hypoplastic vestibular apparatus with poorly developed SCCs, and a lack of coiled cochlear duct (6, 7). Likewise, patients with 22q11.2DS frequently present with anatomic malformations of the middle and inner ears in CT imaging of the temporal bone, primarily malformation of the LSCC with an abnormally small bony island, and incomplete partition type II of the cochlea. With regard to the highly prevalent malformations of the vestibule and LSCC in 22q11.2DS, a recent cross-sectional study found that, although vestibular dysfunctions such as caloric hypofunction and abnormal cervical vestibular-evoked myogenic potentials were common in patients with 22q11.2DS, none had experienced sudden vertigo (4). Postural imbalance has been described in patients with 22q11.2DS, but in most cases, it was associated with general muscle hypotonia or motor delay. Only one case had disequilibrium associated with severe malformations of bilateral LSCCs (8).

Remarkably, our patient presented with Ménière's disease-like symptoms such as whirling-type vertigo with nystagmus, fluctuating hearing loss, and tinnitus. He had bilaterally widened vestibules and LSCCs with an abnormally small bony island, but his VOR functions were spared. These anatomic malformations may have contributed to the development of Ménière's disease-like symptoms in our patient, presumably due to the formation of EH. This hypothesis is supported by the presence of cochlear EH on 3D-FLAIR images, which is concordant with PTA finding showing total deafness in the left ear. Because the patient was maintaining a vertigo-free state with normal VOR functions at the time of MRI examination, vestibular EH might not have been observed. Ménière's disease-like symptoms have been reported in patients with LSCC dysplasia, and histopathologic study revealed EH with hypoplastic endolymphatic sacs (9, 10). MRI evaluation of EH have found enlarged vestibular endolymph in LSCC dysplasia, and a strong negative correlation between the areas of the bony island and the endolymphatic space (11). Likewise, our patient may have had disturbance of endolymphatic flow due to inner ear malformations that caused Ménière's disease-like symptoms. Alternatively, the existence of genetic modifiers interacting with *TBX1* may cause phenotypic variability of audiovestibular dysfunction in 22q11.2DS. A recent study found that non-coding variants in *CRKL* were significantly associated with risk for conotruncal heart defects in individuals with 22q11.2DS (12). Many genes have been related to hereditary non-syndromic hearing loss and Ménière's disease (13–16). It is therefore possible that rare variants in these genes could modify audiovestibular phenotype by interacting with *TBX1* in 22q11.2DS.

Even though it is a rare occurrence, the case described herein shows that acute vertigo with SNHL can be one of the audiovestibular presentations in 22q11.2DS.

## Data Availability Statement

The raw data supporting the conclusions of this article will be made available by the authors, without undue reservation.

## Ethics Statement

All experiments followed the tenets of the Declaration of Helsinki, and informed consents were obtained after the nature and possible consequences of this study had been explained to the participants. This study was approved by the institutional review boards of Pusan National University Yangsan Hospital.

## Author Contributions

K-DC conducted the interpretation of the data and wrote the manuscript. J-YK, S-YC, EO, H-ML, and JR contributed to the interpretation and analysis of the data. J-HC conducted the design, conceptualized the study, interpreted the data, and revised the manuscript. All authors contributed to the article and approved the submitted version.

## Conflict of Interest

The authors declare that the research was conducted in the absence of any commercial or financial relationships that could be construed as a potential conflict of interest.
